# Micro- and nanometric characterization of the celestite skeleton of acantharian species (Radiolaria, Rhizaria)

**DOI:** 10.1038/s41598-022-06974-2

**Published:** 2022-02-18

**Authors:** Rina Fujimaki, Noritoshi Suzuki, Katsunori Kimoto, Yukiko Nagai, Yuya Oaki, Shinji Shimode, Takashi Toyofuku, Hiroaki Imai

**Affiliations:** 1 Department of Applied Chemistry, Faculty of Science and Technology, Keio University, 3-14-1 Hiyoshi, Kohoku-ku, Yokohama, 223-8522 Japan; 2 Department of Earth Science, Graduate School of Science, Tohoku University, 6-3, Aoba, Aramaki, Aoba-ku, Sendai, 980-8578 Japan; 3 Research Institute for Global Chang (RIGC), Japan Agency for Marine-Earth Science and Technology (JAMSTEC), Natsushima-cho 2-15, Yokosuka, 237-0061 Japan; 4 X-Star, Japan Agency for Marine-Earth Science and Technology (JAMSTEC), Natsushima-cho 2-15, Yokosuka, 237-0061 Japan; 5 Manazuru Marine Center for Environmental Research and Education, Graduate School of Environment and Information Sciences, Yokohama National University, 61 Iwa, Manazuru, 259-0202 Japan

**Keywords:** Structural biology, Materials science

## Abstract

We clarified the specific micrometric arrangement and nanometric structure of the radiolarian crystalline spines that are not a simple single crystal. A body of the celestite (SrSO_4_) skeleton of acantharian *Acanthometra* cf. *multispina* (Acanthometridae) composed of 20 radial spines having four blades was characterized using microfocus X-ray computed tomography. The regular arrangement of three types of spines was clarified with the connection of the blades around the root of each spine. The surface of the spines was covered with a chitin-based organic membrane to prevent from dissolution in seawater. In the nanometric scale, the mesocrystalline structure that consists of nanoscale grains having distorted single-crystal nature was revealed using scanning- and transmission electron microscopies, electron diffraction, and Raman spectroscopy. The acantharian skeletons have a crystallographically controlled architecture that is covered with a protective organic membrane. These facts are important for penetrating the nature of biogenic minerals.

## Introduction

In nature, organisms produce various inorganic materials with precisely controlled morphologies from a limited selection of ubiquitous elements, such as calcium, silicon, carbon, and oxygen, under ambient conditions. Generally, morphological design is a critically important aspect of biological mineralization processes with regard to the emergence of specific functions. Celestite (SrSO_4_) is remarkably observed as a skeleton of Acantharia, a marine unicellular holoplanktonic protist. Since the utilization of celestite as a skeleton is exclusively known in acantharians in the living world, the structure and property of biological celestite are attracting attention in the fields of biology and material science. In the present study, we characterized the micrometric morphology and nanometric structures of the celestite skeleton of the acantharian species to provide an essential information for clarification of the specific biological crystal.

Acantharia species are identified based on their skeletal architecture, cytological structure and characters of algal symbionts^1,2^. The examined specimens are *Acanthometra* cf. *multispina* Müller (Acanthometridae, Clade F3) and *Phyllostaurus siculus*^3^ (Acanthostauridae, Clade F3)^1^. In Clade F, the celestite skeletons consist of 20 radial spines that geometrically extend from a central point. This central point is constructed by tightly connected fletching roots of the 20 radial spines^1^. These fletching roots are combined with cytologic fibers named “myoneme”^4^. As these radial spines are embedded in the cytoplasmic membrane, they are endoskeletons. We can observe a particularly ordered spatial arrangement of spines for the acantharian skeleton.

The geometric arrangement of radial spines is called Müller’s law^5^ and is composed of two quartets of polar radial spines alternating with two quartets of tropical radial spines and one quartet of equatorial radial spines^1,3,5^. The biology of Müller’s law is poorly understood, although several models have been proposed^6,7^. The crystal structure of celestite was investigated using X-ray diffraction^8^, electron diffraction, and transmission electron microscopy (TEM) techniques^9^. These papers concluded that each spine is a single crystal of celestite with a specific crystallographic orientation, but uncertainty still remains regarding the spine arrangement of the celestite skeleton of the selected acantharian specimens, which was characterized using various techniques, including microfocus X-ray computed tomography (CT), TEM, scanning electron microscopy (SEM), and Raman scattering spectrometry.

Micrometric and nanometric studies on acantharean skeletons are needed to understand the specific nature of the biogenic products. Biominerals have been revealed to have hierarchical architectures that are built up of nanoscale grains incorporated with organic polymers, regardless of their polymorph^10–16^. The specific crystal structure consisting of nanometric building units aligned in the same crystallographic orientation is called mesocrystal. The specific mesoscopic textures provide the excellent mechanical properties of biominerals. Thus, studies of hierarchical architectures of various biominerals would contribute to the development of emergent materials^17–23^.

The present article focuses on the micrometric and nanometric morphologies of the celestite skeleton of *A*. cf. *multispina*. Our research achieved an accurate description of the specular arrangement and the mesocrystal nature of biological celestite. Here, we study the essence of biological crystals as a skeleton of marine protists.

## Results and discussion

### Arrangement of spines

**Figure 1:**
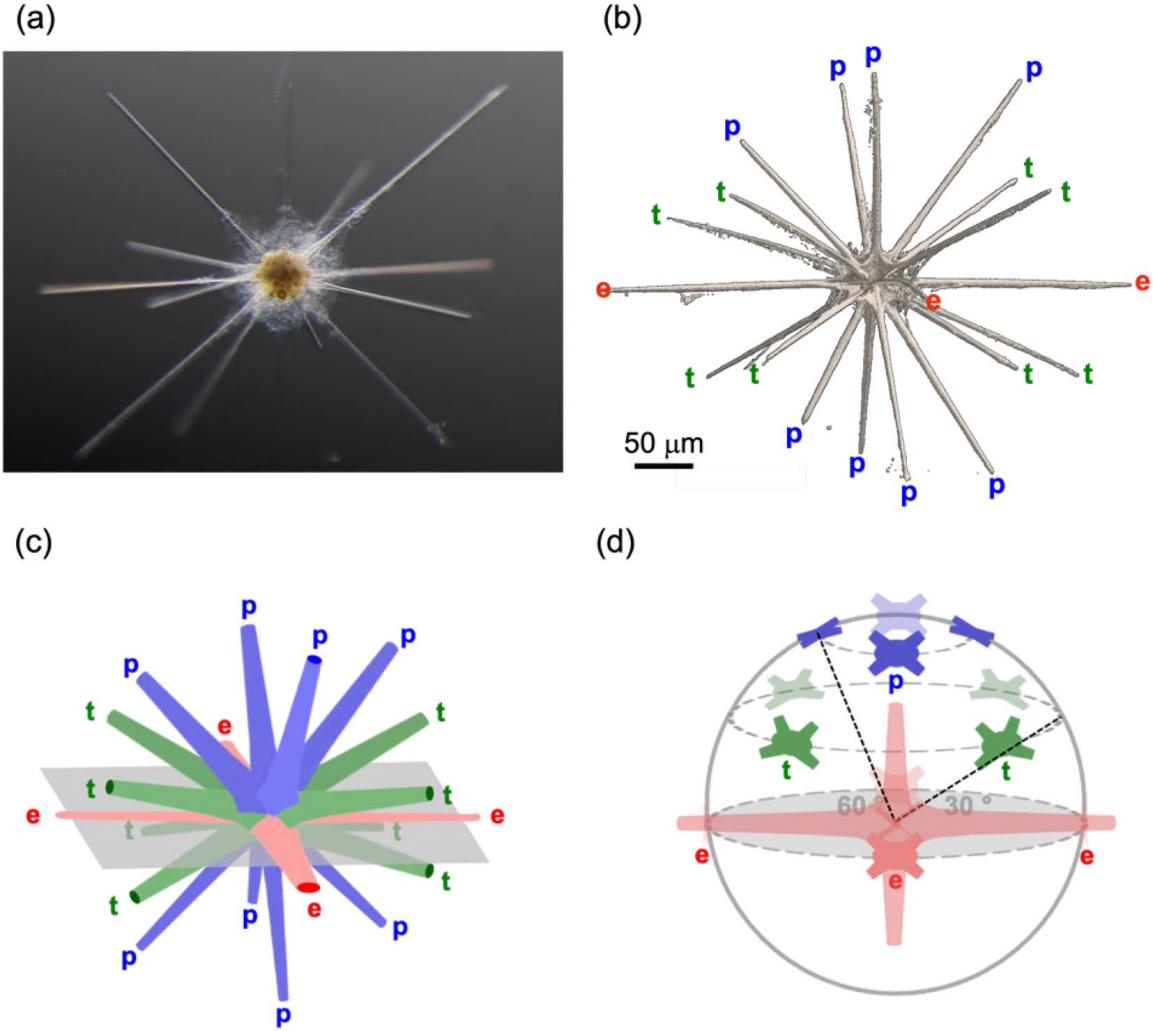
Spine arrangement of *Acanthometra* cf. *multispina* (Acanthometridae). (**a**) Optical microscope image [fully grown individual], (**b**) microfocus X-ray CT image, (**c**) schematic of the 20 spines, and (**d**) orientation of spines in the upper hemisphere of the unit sphere. Red e: equatorial spines, green t: tropical spines, blue p: polar spines, and gray: the equatorial plane.

Figure 1 shows an optical microscope image and a microfocus X-ray CT image of a whole skeleton of *Acanthometra* cf. *multispina.* There are 20 radial spines (“spine” hereafter) radiating from the skeletal center, as illustrated in Fig. 1c. The arrangement of four equatorial spines (e), four diametric (= 8) tropical spines (t), and four diametric (= 8) polar spines (p) agrees with Müller’s law^5^ (Fig. 1b). Given a unit sphere (Fig. 1d) whose origin is defined at the skeletal center and whose x- and y-axes are designed to the equatorial spines, the deviation angles of tropical and polar spines from the equatorial plane are 30° and 60°, respectively. The equatorial plane is defined by the plane with x- and y-axes.

**Figure 2:**
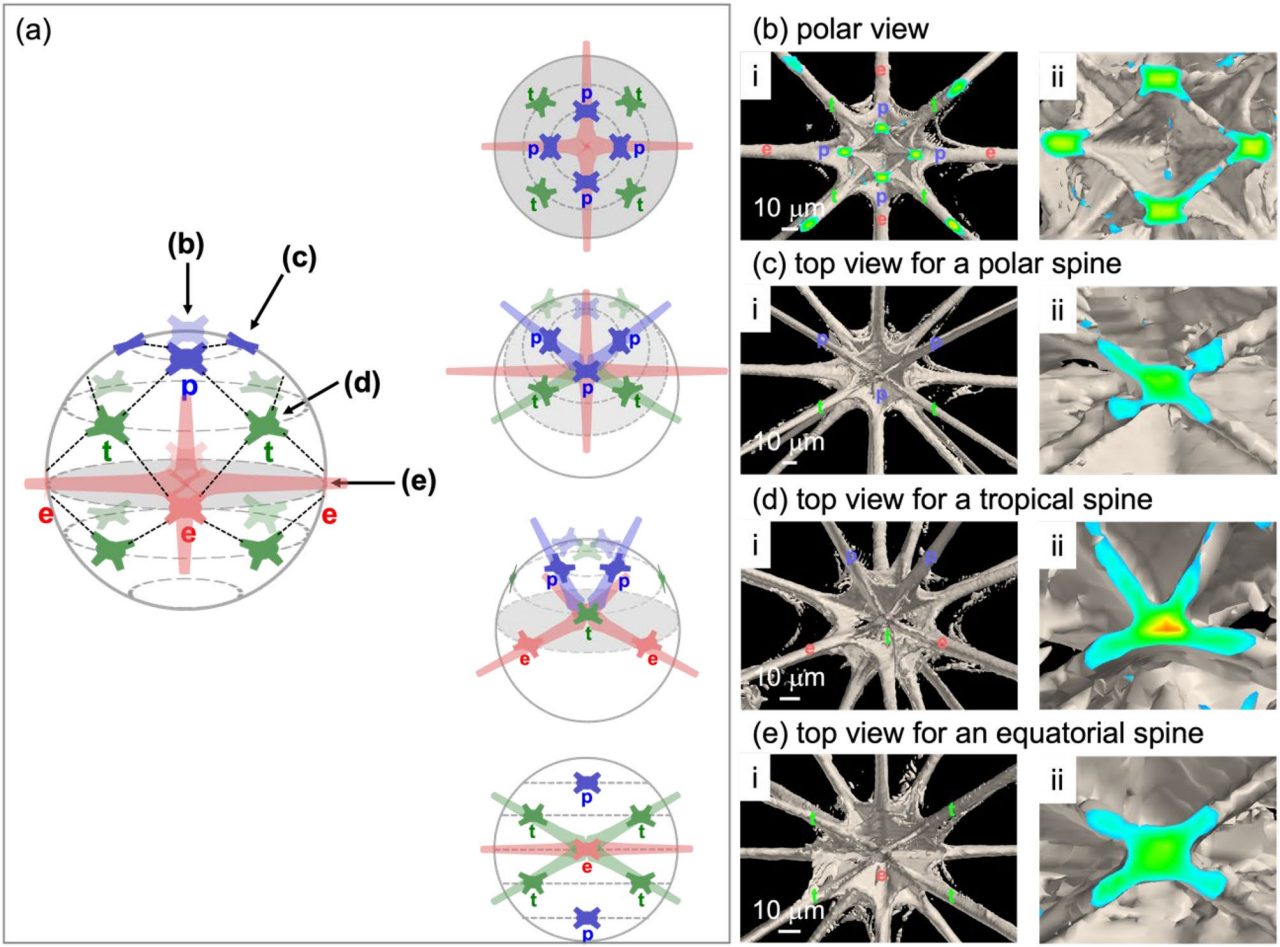
(**a**) Position of spines under a unit sphere, (**b-i**, **c-i**, **d-i**, **e-i**) correspondences of spine positions to the real images and (**b-ii**, **c-ii**, **d-ii**, **e-ii**) their enlarged CT images. (**b**) Polar view and top view for (**c**) polar, (**d**) tropical, and (**e**) equatorial spines. Red e: equatorial spines, green t: tropical spines, blue p: polar spines, and gray: the equatorial plane.

A more detailed spine arrangement is displayed in the enlarged microfocus X-ray CT images (Fig. 2). On the polar-view images (Fig. 2b), four equatorial spines and four diametric (= 8) polar spines are at the same positions with fourfold rotational symmetry, and four diametric (= 8) tropical spines are located at intermediate positions. All the spines have four blades around their root and connected through the blades (Fig. 2b-ii). From the top views of the spines, we characterized the connecting modes of the spines around the skeletal center. A polar spine is connected to two other polar and two tropical spines (Fig. 2c). A tropical spine is linked to two polar and two equatorial spines (Fig. 2d). An equatorial spine is directly attached to four tropical spines (Fig. 2e). The connecting angles around the equatorial and polar spines are almost the same with a twofold rotational symmetry (Fig. 2c, e). The arrangement of the four blades agrees with that reported in a previous study^7^. On the other hand, the blades of a tropical spine are arranged in a mirror symmetry (Fig. 2d).

### Micrometric morphology and structure of spines

**Figure 3:**
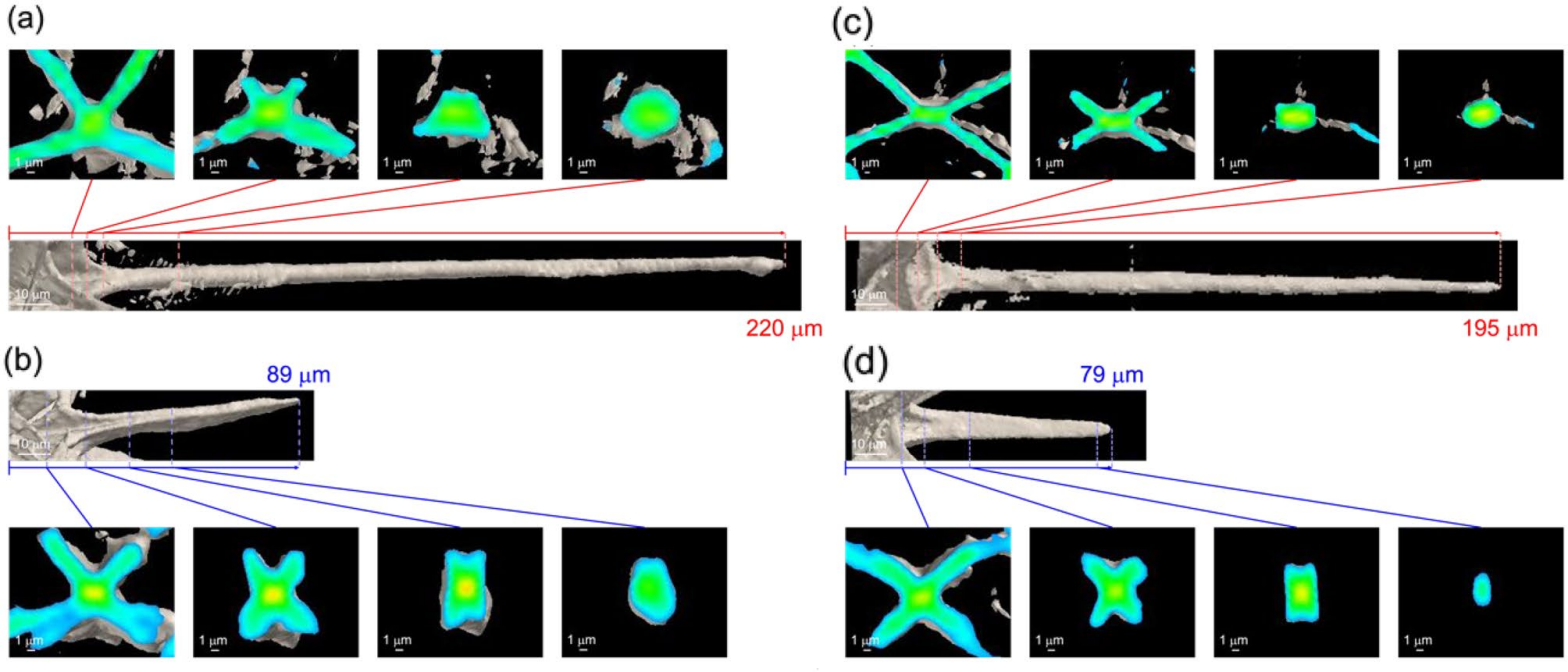
Cross-sectional microfocus CT images of (**a**, **b**) the tropical and (**c**, **d**) polar spines of (**a**, **c**) a large specimen and (**b**, **d**) a small specimen. The images of whole specimens and the cross-sectional images of equatorial spines are shown in Fig. S1 in the SI.

We revealed the morphology of a spine from several cross-sectional images produced by microfocus X-ray CT. Figure 3 shows cross sections of the polar and tropical spines of small and large specimens. Basically, the shape of the cross section of a spine is rectangular around the root but ellipsoidal in the remaining parts, including the distal end. As mentioned above, four blades are attached around their root. By comparing the fletching root of a small specimen with a large specimen, the bladed parts are roughly equal in size regardless of the different total lengths of the spines. This suggests that the spines elongate from the distal end with ontogenetic growth. The equatorial spines are 1.1–1.3 times longer than the tropical and polar spines (Table S1 in the Supporting Information (SI)).

**Figure 4:**
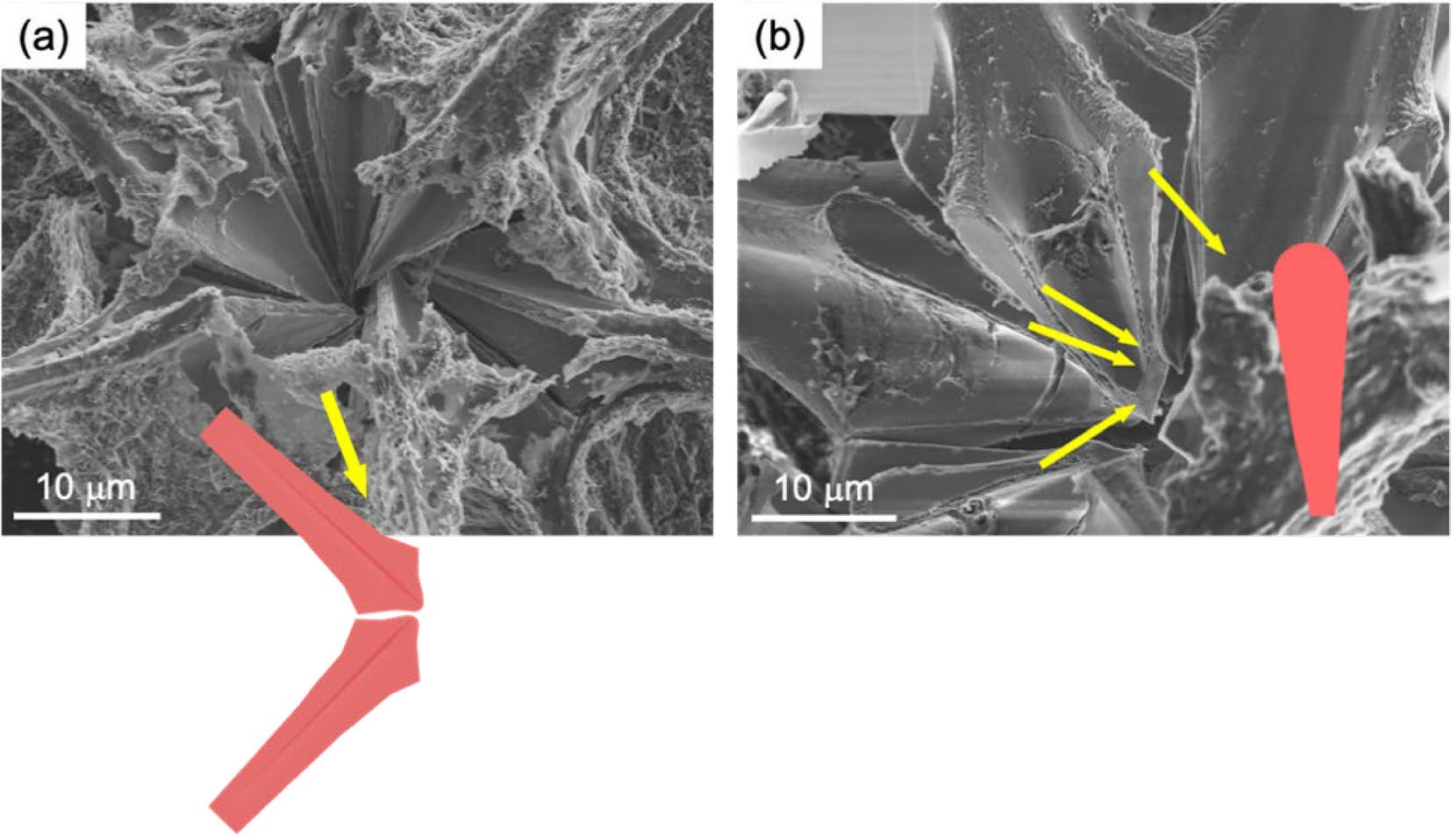
(**a**, **b**) SEM images of the fletching roots of the spines in a broken specimen.

Figure 4 exhibits SEM images of the partly broken central part, showing the connection among the fletching-roots of the spines. The spines with blades are found to be separated at the skeletal center. This indicates that the spines are indirectly connected through the blades. The junction planes of the blades are teardrop-shaped.

**Figure 5:**
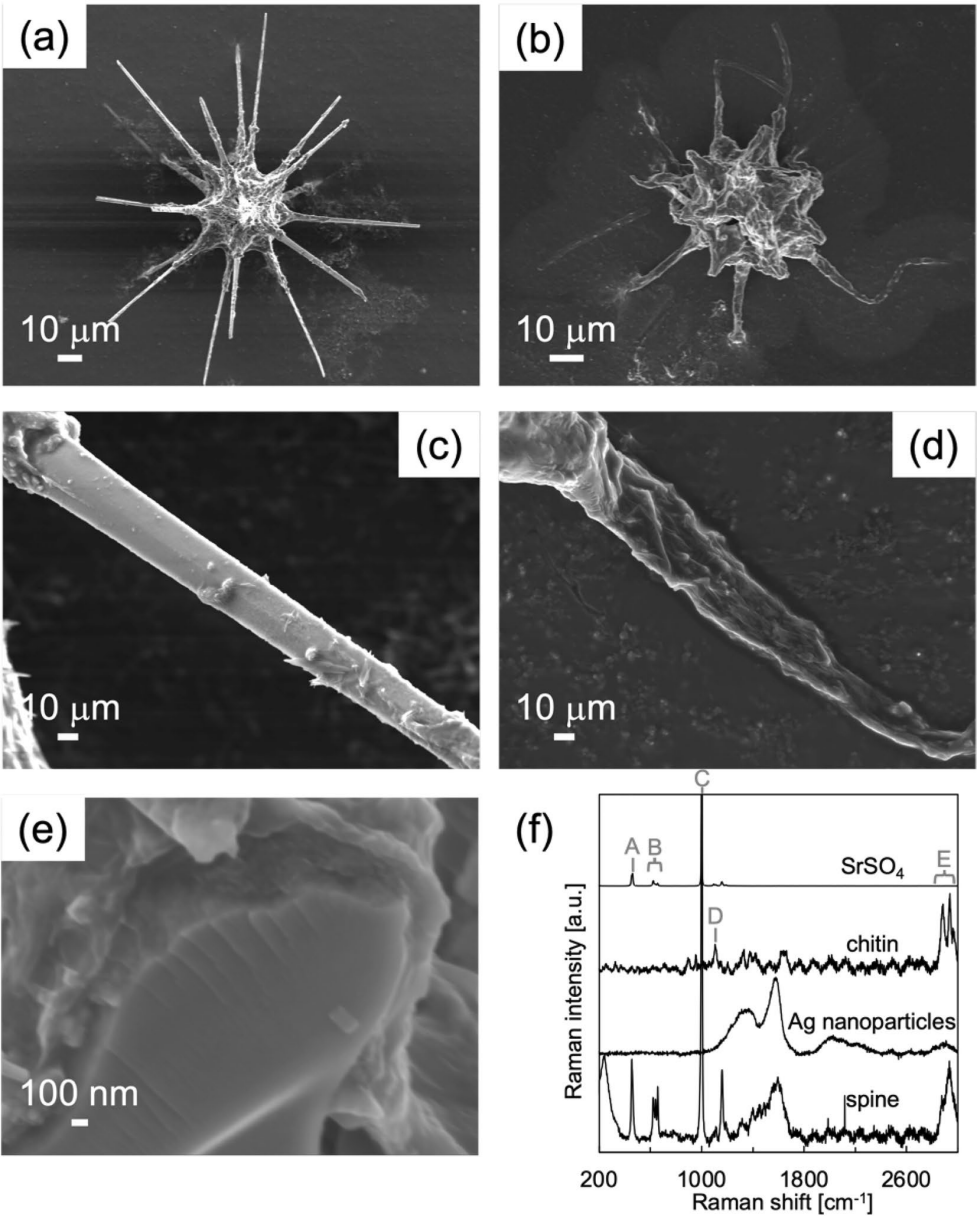
SEM images (**a**, **c**) before and (**b**, **d**) after immersion in an EDTA solution. (**e**) SEM image of a cross section of a broken spine before EDTA treatment. (**f**) Raman spectra of spine, celestite, standard chitin, and silver particles. The Raman signal for the spine was enhanced by the surface plasmon of silver nanoparticles attached to the specimen. The Raman signals A, B, and C are assigned to the S–O symmetric stretching vibration, the S–O double expansion and stretching vibration, and the S–O asymmetric stretching vibration, respectively^24^. The Raman signals D and E are assigned to C–O–C and C-O stretching vibrations and to a C-H stretching vibration, respectively^25^.

We immersed the skeletons in an aqueous solution containing 250 mmol/dm^3^ ethylenediaminetetraacetic acid (EDTA) to dissolve inorganic crystals. Figure 5 is the specimens before and after the EDTA treatment. By using EDTA, skeletons collapsed upon immersion. The removal of the solid skeleton by this treatment was confirmed by elemental analysis using an energy-dispersive X-ray spectrometer (EDS) (Fig. S2 in the SI). This indicates the dissolution of celestite in the skeleton. After dissolution of the solid skeleton, we confirmed an organic membrane covering the spines. From an SEM image of a cross section of a broken spine (Fig. 5e), the thickness of the membrane is estimated to be approximately 100 nm. We characterized the organic membranes with a deposition of silver particles to observe surface-plasmon-enhanced Raman spectra (Fig. 5f) and Calcofluor White Stain (Sigma Aldrich) that binds with cellulose and chitin contained cell walls. According to the Raman spectra, the membranes are deduced to be mainly composed of chitin ((C_8_H_13_O_5_N)_*n*_). Moreover, we confirmed that the spines yielded fluorescence after addition of one drop of Calcofluor White Stain (Fig. S3 in the ESI). Thus, the spines are enveloped by a chitin-based organic membrane. As shown in Fig. 5, the chitin-based envelopes collapsed after dissolution of celestite cores. This suggests that the spines are not controlled by the organic membranes as a template.

### Nanometric and crystallographic structures of spines

**Figure 6:**
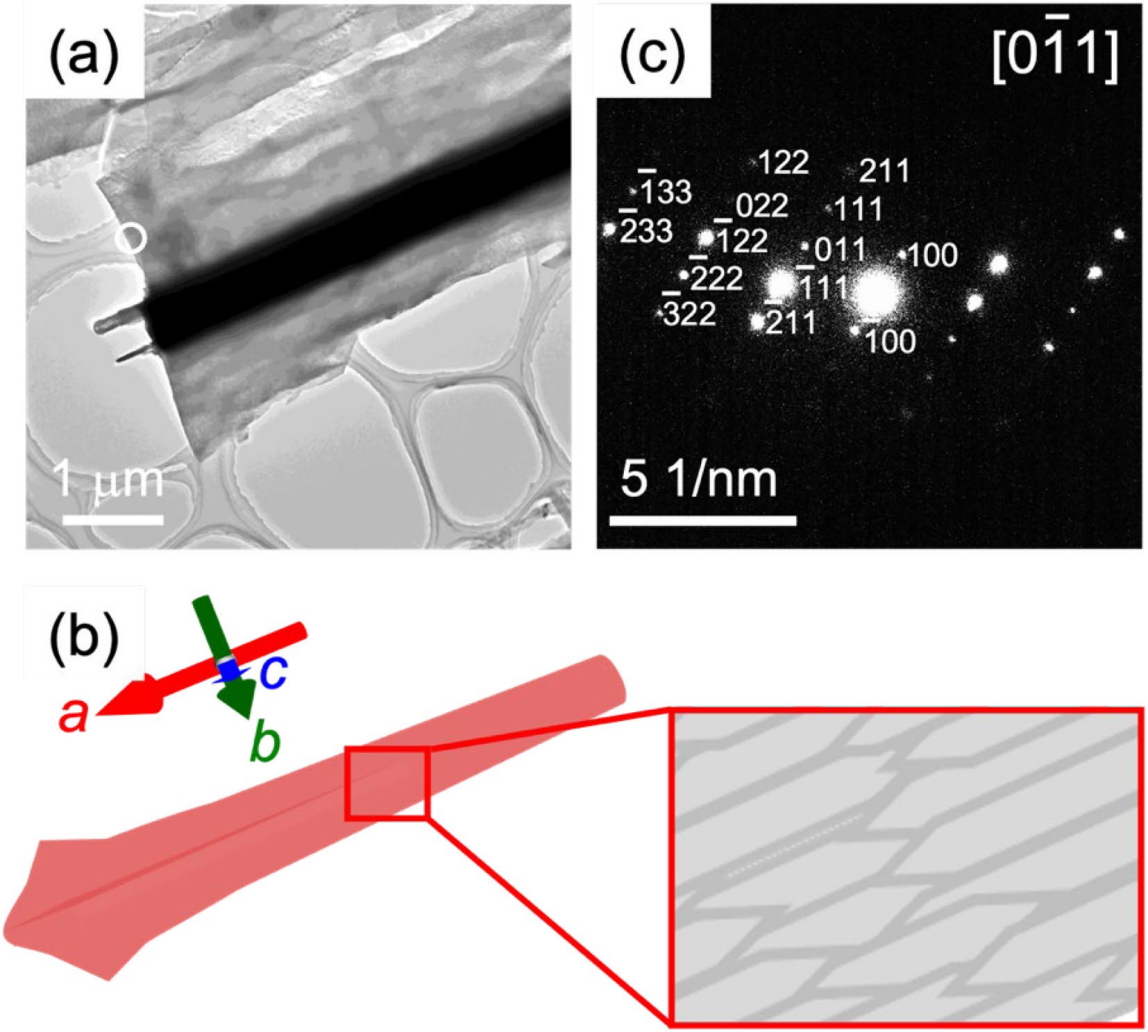
(**a**) A typical TEM image and (**b**) schematic illustration of a bladed spine with (**c**) an SAED pattern.

Figure 6 shows SEM and TEM images of spines with a typical SAED pattern. From the diffraction spots, the spines are assigned to celestite that is elongated in the *a*-axis direction and that has a single-crystal nature. The blades are suggested to expose the {110} planes. These facts about the crystallographic structure are in agreement with the assignment in a previous work^26^.

**Figure 7:**
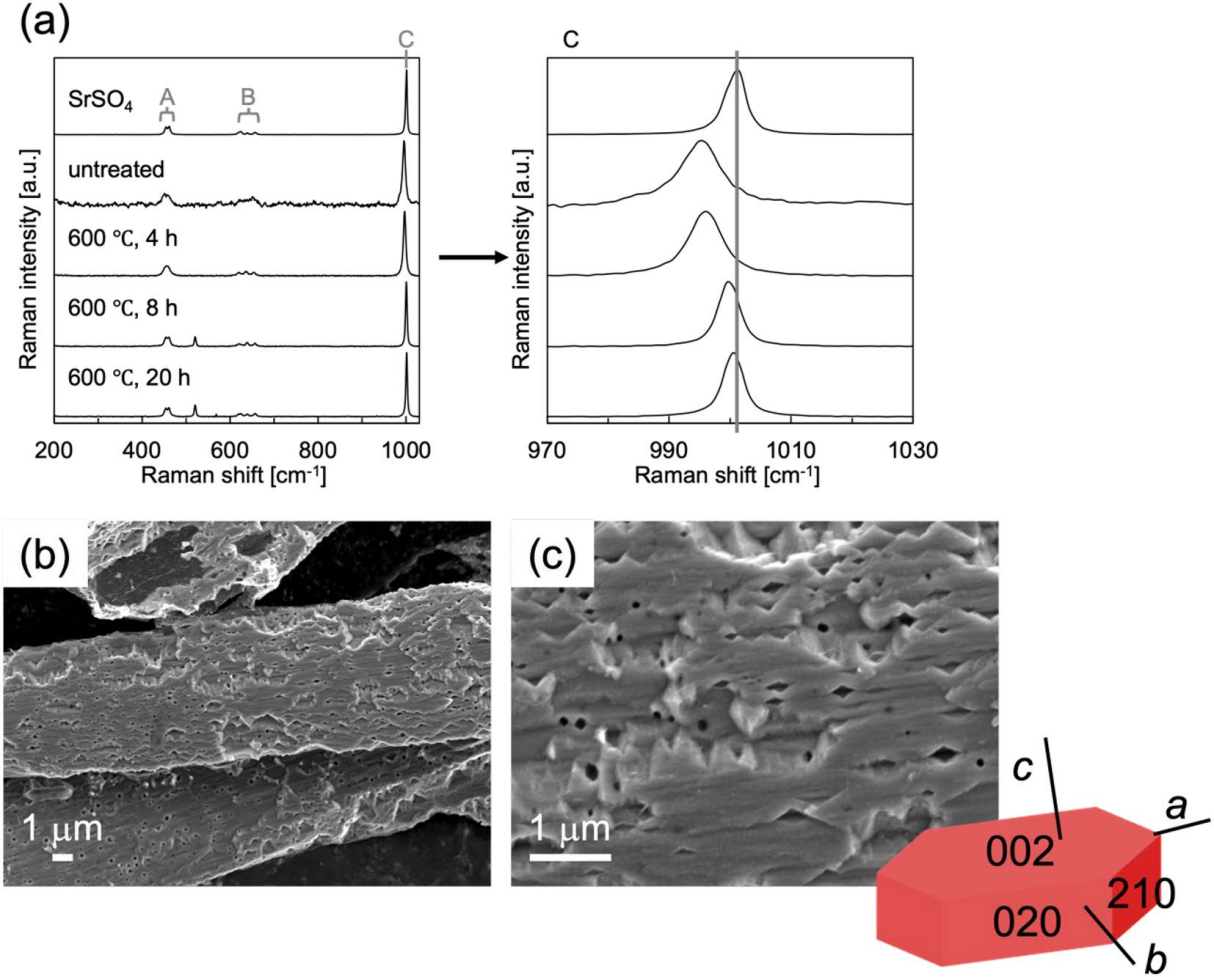
(**a**) Raman spectra of spines before and after calcination at 600 °C in air and (**b**, **c**) SEM images of the spines after calcination at 600 °C for 4 h in air and subsequent etching with pure water for 2 h. The Raman signals A, B, and C are assigned to the S–O symmetric stretching vibration, the S–O double expansion and stretching vibration, and the S–O asymmetric stretching vibration, respectively^25^.

The spines were also assigned to celestite using Raman spectroscopy (Fig. 7a). Interestingly, however, we observed a slight shift in the signal due to the S–O asymmetric stretching vibration to a lower wavenumber. This suggests the presence of the lattice strain of a celestite crystal in the skeleton. The strain was recovered after calcination at 600 °C for 20 h in air. Figure 7b, c shows SEM images of spines after removal of the organic matter by calcination at 600 °C for 4 h in air and subsequent etching with pure water for 2 h. Although the associated organic matter was removed with mild calcination for 4 h, the strain remained in the crystalline lattice. We observed fibrous units ∼ 100 nm wide on the spine surface. Tilted faces are assignable to the (210) plane by comparing the shape of artificially produced celestite crystals^27^. These results suggest that the spines are not a homogeneous single crystal but a bundle of fibrous units elongated in the *a*-axis direction. Finally, we conclude that the acantharian skeleton is composed of a celestite mesocrystal consisting of nanoscale units that are arranged in the same crystallographic orientation.

As shown in Fig. S3 in the SI, we succeeded in producing celestite mesocrystals consisting of fibrous units. The bundled structure was formed through precipitation in a supersaturated solution containing poly(acrylic acid). The fibrous units that are elongated in the *a*-axis direction are arranged in the same orientation. The lattice strain of the artificial celestite mesocrystal is similar to that of the acantharian spines. Since the organic content of the products was estimated to be ca. 3 wt%, almost the same amounts of organic molecules are deduced to be included in the biological celestite.

## Conclusion

The macroscopic arrangement, micrometric morphology, and nanometric structures of the celestite (SrSO_4_) skeleton of acantharian *Acanthometra* cf. *multispina* (Acanthometridae) were completely characterized using various techniques. Here we clarified the specific micrometric arrangement and nanometric structure of the radiolarian crystalline spines that are not a simple single crystal. Three types of spines covered by a chitin-based organic membrane were regularly arranged with the connection of the wings around the center of the skeleton. The celestite spines have a mesocrystalline structure that consists of nanoscale grains having a distorted single-crystal nature. Finally, the acantharian skeletons were found to have a crystallographically controlled architecture that is covered with a protective organic membrane. The specific architecture would provide characteristic properties of the biogenic products.

## Experimental

Plankton samplings were conducted at 35° 09.45′ N, 139° 10.00′ E in the western part of Sagami Bay in southern Japan on R/V Tachibana of the Manazuru Marine Center for Environmental Research and Education, Yokohama National University. Individuals of acantharians were collected by plankton nets (diameter: 80 cm, side length: 3 m, mesh size: 100 μm or diameter: 45 cm, side length: 1.8 m, mesh size: 180 μm). The living specimens were immersed in deionized water, and water freeze-drying equipment (FD-6500; Kyowa Corporation) was used to obtain freeze-dried samples. Cross sections of the dried samples exposed by crushing were observed by microfocus X-ray CT, SEM and optical microscopy. Shell morphometry of acantharian was performed by microfocus X-ray CT (ScanXmateD160TSS105, Comscantechno Co., Ltd.) equipped in Japan Agency for Marine-Earth Science and Technology (JAMSTEC). A high-resolution setting (X-ray focus diameter: 0.8 µm; X-ray tube voltage: 90 keV; X-ray tube current: 37 µA; detector array size of 1024 × 1024 pixels; 2000 projections in 360° rotations) was applied. The geometric resolution of the isotropic voxel size was from 0.28 to 0.46 µm/voxel. We used the ConeCTexpress (White rabbit Corp.) software for correction and reconstruction tomography data and the general principle of Feldkamp cone beam reconstruction was followed to reconstruct image cross sections based on filtered back projections. The surfaces and cross sections of the samples were coated with osmium for detailed observation using a scanning electron microscope (SEM, FEI Helios G4 UX, JEOL JSM-7100) operated at 2.0–15.0 kV. The compositions were identified using Raman scattering spectroscopy and energy-dispersive X-ray analysis (JEOL JED-2300). Micro-Raman spectroscopy was performed using a laser confocal microscope (inVia, Renishaw). The 532 nm excitation laser was focused on the sample surface with a 100 × objective of the microscope. The size of the laser spot was approximately 1 μm in diameter. Chitin standard ((C_8_H_13_O_5_N)_*n*_) was purchased from Kanto Chemical. Crystalline parts in the spines were characterized by transmission electron microscopy (TEM, FEI Tecnai G2). The samples were dropped with water on a copper grid and crushed with a needle to release crystalline parts from the main body. A suspension containing crystals was quickly dried for a few minutes on a copper grid for TEM observation. Crystalline parts were dissolved to observe the frameworks by immersing specimens in deionized water for several hours. After a series of these treatments, the examined specimens are taxonomically identified under the modern classification concept and named following the International Code of Zoological Nomenclature.

## Supplementary Information

Supplementary Information.
