# Supplementary material for: Micro- and nanometric characterization of the celestite skeleton of acantharian species (Radiolaria, Rhizaria)
Source: Sci Rep. 2022 Feb 18;12:2848. doi: 10.1038/s41598-022-06974-2 (PMC8857179; doi:10.1038/s41598-022-06974-2)
Supplement: Supplementary file 1 — Supplementary Information. [file 41598_2022_6974_MOESM1_ESM.pdf]

Supplementary Information

Micro- and nanometric characterization of the celestite skeleton of acantharian species (Radiolaria, Rhizaria)

Rina Fujimaki[1] • Noritoshi Suzuki[2] • Katsunori Kimoto[3] • Yukiko Nagai[4] • Yuya Oaki[1] • Shinji Shimode[5] • Takashi Toyofuku[4] • Hiroaki Imai[1*]

Address

1: Department of Applied Chemistry, Faculty of Science and Technology, Keio University, 3-14-1 Hiyoshi, Kohoku-ku, Yokohama 223-8522, Japan /E-mail※: hiroaki@applc.keio.ac.jp

2: Department of Earth Science, Graduate School of Science, Tohoku University, 6-3, Aoba, Aramaki, Aoba-ku, Sendai 980-8578, Japan

3: Research Institute for Global Change (RIGA), Japan Agency for Marine-Earth Science and Technology (JAMSTEC), Natsushima-cho 2-15, Yokosuka 237-0061, Japan

4: X-star, Japan Agency for Marine-Earth Science and Technology (JAMSTEC), Natsushima-cho 2-15, Yokosuka 237-0061, Japan

5: Manazuru Marine Center for Environmental Research and Education, Graduate School of Environment and Information Sciences, Yokohama National University, 61 Iwa, Manazuru 259-0202, Japan.

[*]Corresponding authors

E-mail address: hiroaki@applc.keio.ac.jp

Figure S1

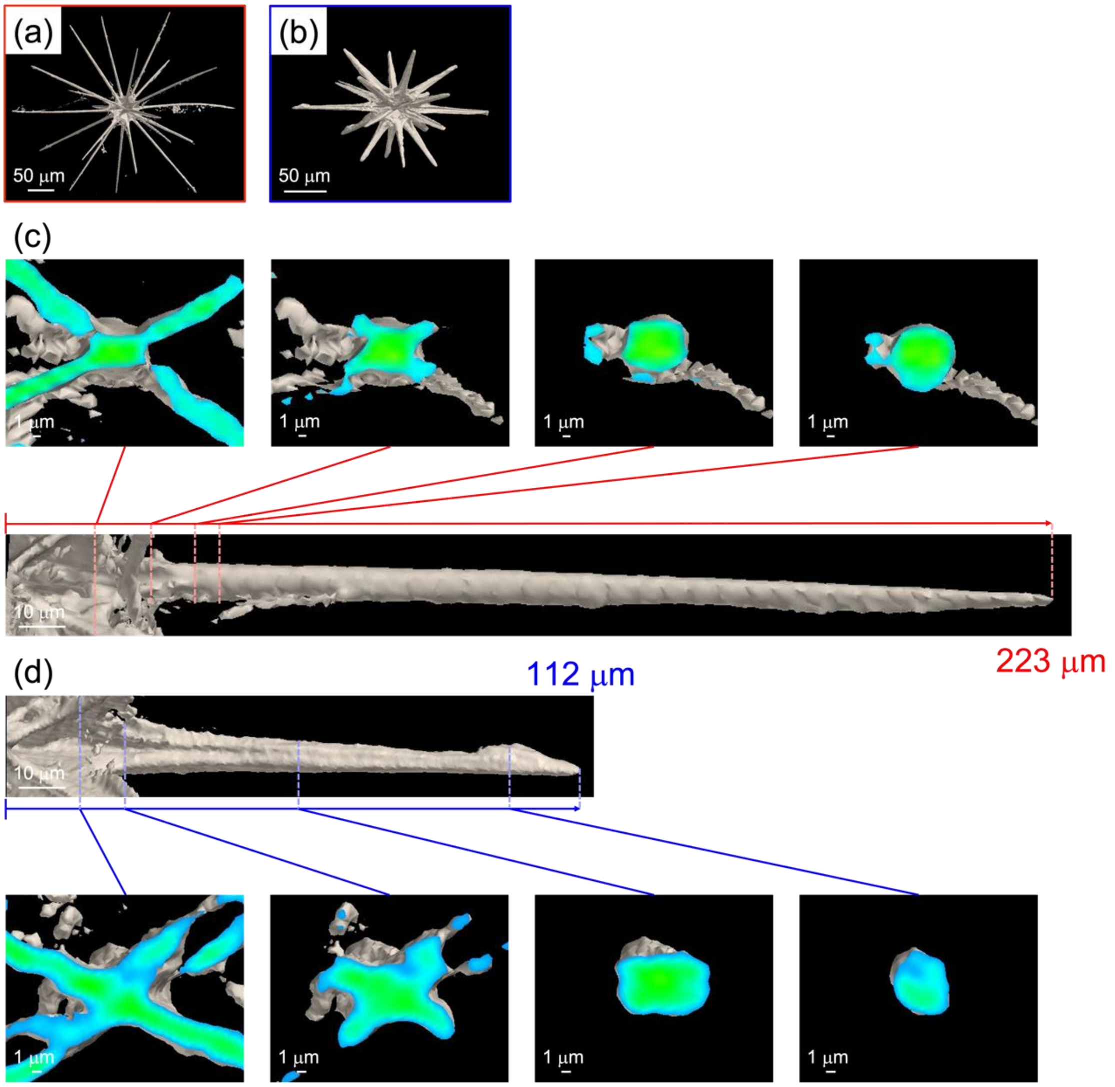

Supplementary Figure 1. (a, b) The whole microfocus CT images. Cross-sectional microfocus CT images of equatorial spines of (c) a large individual and (d) a small one.

Figure S2

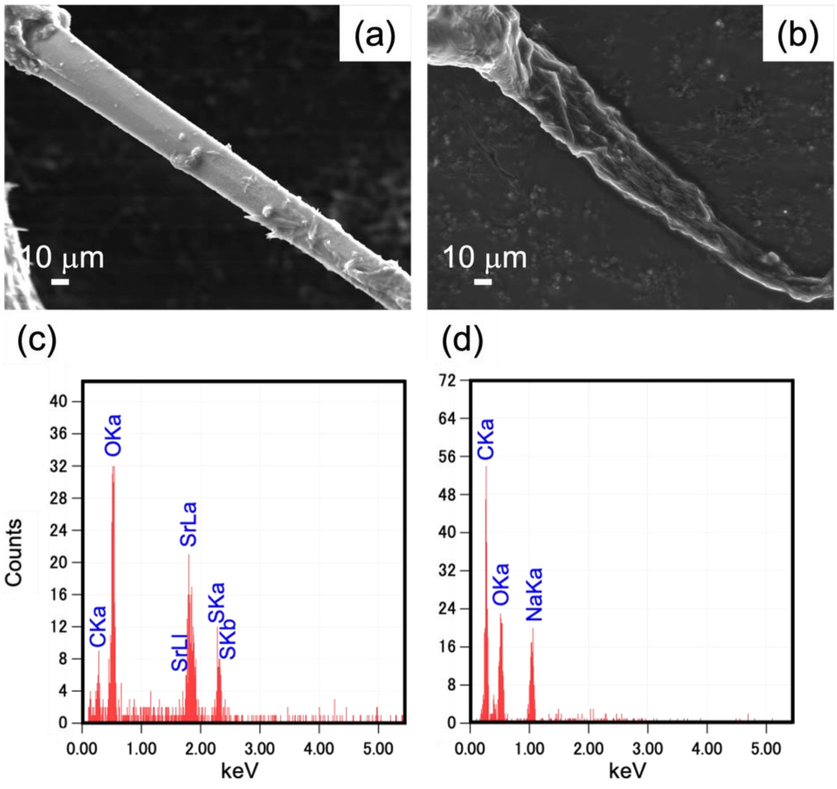

Supplementary Figure 2. (a, b) SEM images with (c, d) EDS spectra (a, c) before and (b, d) after immersion into an EDTA solution.

Figure S3

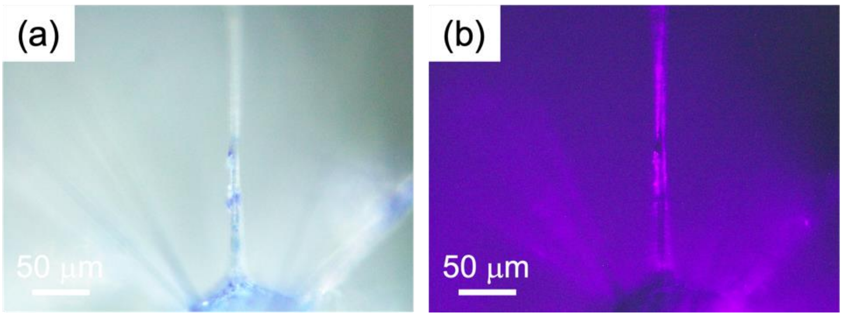

Supplementary Figure 3. Fluorescence microscope images of spines stained with Calcofluor White Stain (Sigma Aldrich) (a) under natural light and (b) ultraviolet illumination (400 nm). Calcofluor White Stain is non-specific fluorochrome that binds with cellulose and chitin contained cell walls. We confirmed that spines yielded fluorescence after addition of one drop of Calcofluor White Stain.

Figure S4

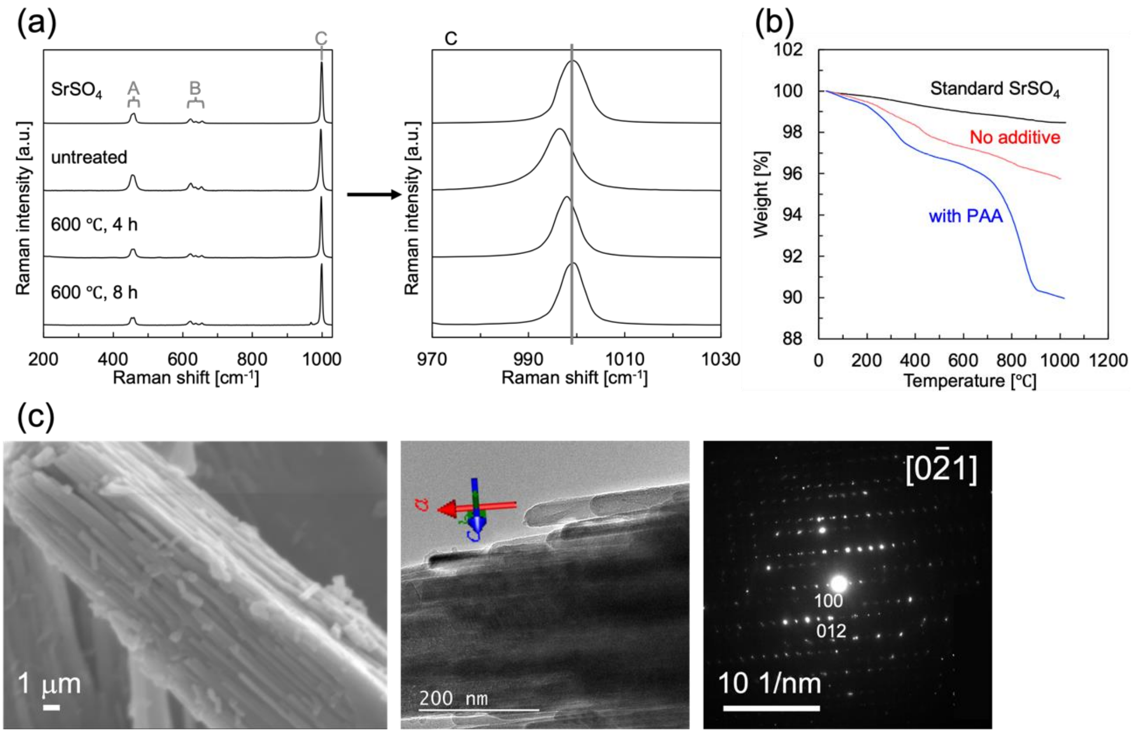

Supplementary Figure 4. (a) Raman spectra of artificailly produced celestite with PAA before and after calcination at 600°C in air. (b) Thermogravimetry (TG, Shimadzu DTG-60) was performed to evaluate the organic content. TG curves of artificailly produced celestite with and witout PAA. (c) SEM and TEM images with an SAED pattern of artificailly produced celestite mesocrystal with PAA. Raman signals A, B, and C are assigned to the S-O symmetric stretching vibration, the S-O double expansion and stretching vibration, and the S-O asymmetric stretching vibration, respectively.

We synthesized celestite using the following procedure. Solutions of Na_2SO_4 (1.0 mol/dm^3) and of $SrCl_2$ (1.0 mol/dm^3) were prepared by dissolving analytically pure Na_2SO_4 and $SrCl_2 \cdot 6H_2O$ into purified water. A certain amount (0.01 wt%) of PAA was dissolved into the $SrCl_2$ solution under stirring. The Na_2SO_4 solution was added into the mixed aqueous of $SrCl_2$ and PAA under vigorous stirring at room temperature. After stirring for 10 min, the suspension was kept for 24 h. We recovered precipitates by filtration and subsequent rinse with ethanol.
